# Infer Thermal Information from Visual Information: A Cross Imaging Modality Edge Learning (CIMEL) Framework

**DOI:** 10.3390/s21227471

**Published:** 2021-11-10

**Authors:** Shuozhi Wang, Jianqiang Mei, Lichao Yang, Yifan Zhao

**Affiliations:** 1 School of Aerospace, Transport and Manufacturing, Cranfield University, Bedford MK43 0AL, UK; shuozhi.wang@cranfield.ac.uk (S.W.); lichao.yang@cranfield.ac.uk (L.Y.); 2 School of Electronic Engineering, Tianjin University of Technology and Education, Tianjin 300222, China; meijianqiang@tute.edu.cn

**Keywords:** image enhancement, edge detection, deep learning, thermography

## Abstract

The measurement accuracy and reliability of thermography is largely limited by a relatively low spatial-resolution of infrared (IR) cameras in comparison to digital cameras. Using a high-end IR camera to achieve high spatial-resolution can be costly or sometimes infeasible due to the high sample rate required. Therefore, there is a strong demand to improve the quality of IR images, particularly on edges, without upgrading the hardware in the context of surveillance and industrial inspection systems. This paper proposes a novel Conditional Generative Adversarial Networks (CGAN)-based framework to enhance IR edges by learning high-frequency features from corresponding visual images. A dual-discriminator, focusing on edge and content/background, is introduced to guide the cross imaging modality learning procedure of the U-Net generator in high and low frequencies respectively. Results demonstrate that the proposed framework can effectively enhance barely visible edges in IR images without introducing artefacts, meanwhile the content information is well preserved. Different from most similar studies, this method only requires IR images for testing, which will increase the applicability of some scenarios where only one imaging modality is available, such as active thermography.

## 1. Introduction

Infrared (IR) is a kind of electromagnetic radiation with a longer wavelength than that of visible light. Infrared thermography has been widely used in different fields, such as monitoring [1], medicine [2], psychophysiology [3], nondestructive testing [4] (NDT) and so forth.

Although significant progress has been achieved in IR imaging, the spatial resolution is still one of the major limiting factors and bottlenecks for industrial thermography applications, mainly due to the high-cost of sensors. Typically, the pixel dimension of thermography is 640×480, which is relatively low compared with modern RGB photography. Although there are some high-end IR cameras with improved spatial-resolution, these cameras are usually much more expensive. Furthermore, even with the same spatial-resolution, the boundary of objects in thermal images is not as sharp as that in digital images. Viewing from the imaging principle: the digital imaging system typically obtains images by applying CCD or CMOS sensors, based on the difference in the intensity of light in the range of 0.4–0.7 μm reflected by the surface of the observed target, with high contrast and improved resolution. While infrared thermal imaging technology is based on receiving radiant energy with longer wavelengths in the range of 3–12 μm. Due to the difference in minimum resolvable temperature difference between the object and the background, together with the distance that it is measured from, the target is quickly submerged in the dark background. This phenomenon is likely to lead to the blurring effect of the acquired IR images. This is particularly problematic in active thermography, where the boundary of damage in thermal images could be blurred, leading to less accurate damage measurement and reduced reliability.

### 1.1. Non-Deep Learning Based Approaches

Classic image processing methods dominated the edge enhancement for IR images before 2013. Silveman [5] presented a survey of algorithms for the display and enhancement of infrared images, where algorithms were grouped into global monotonic mappings and mappings for local contrast enhancement. As an extendsion of the isotropic smoothing Gaussian pyramid, Acton [6] proposed to utilise Anisotropic Diffusion Pyramid (ADP), created by the successive application of anisotropic diffusion and sub-sampling, to detect and enhance edges in IR images. By adjusting the global data distribution to be equal, Histogram Equalization (HE) related methods were widely applied for IR image enhancement [7,8,9]. Nevertheless, Branchitta et al. [10] combined dynamic-range compression and contrast enhancement techniques to overcome the over-enhancing and compromised detail issues of HE-based methods, which is also referred as Contrast-Limited Adaptive Histogram Equalization (CLAHE). Considering the relatively low signal-to-noise ratio (SNR) characteristics of IR images, some researchers adopted wavelet related algorithms to achieve better noise-reducing performance and edges preserving effects [11,12], while others separated the detail/edge information from the original IR image for different downstream tasks [13,14,15,16,17]. Furthermore, top-hat transform [18], gradient domain [19,20], shearlet domain [21,22] and frequency domain [23] have also been investigated for the IR edge/detail enhancement purpose. Some other related works including an improved unsharp mask algorithm [24], gradient distribution via Cellular Automata [25], morphological operators [26], all-optical upconversion imaging techniques [27], the iterative contrast enhancement method [28], and the gravitational force and lateral inhibition network [29]. Overall, it should be noted that most of the existing non-deep learning IR edge/detail enhancement approaches usually follow the state-of-the-art algorithms from the visual image processing domain.

### 1.2. Deep Learning Based Approaches

Deep learning approaches, particularly Convolutional Neural Network (CNN)-based and Generative Adversarial Networks (GAN)-based methods, have recently shown explosive popularity due to their superior performance in the enhancement of visible-spectrum images and IR images. Choi et al. [30] proposed a thermal image enhancement method based on CNN guided by RGB images, which directly learns an end-to-end mapping from a single low-resolution image to the desired high-resolution image. Lee et al. [31] proposed a convolutional neural network for thermal image enhancement by incorporating the brightness domain with a residual-learning technique for training, which improved the enhancement performance and speed of convergence. In order to enhance the long-range IR images, Fan et al. [32] introduced an approach to predict the target and background by a CNN architecture and the dim IR image was enhanced by amplifying the targets and subtracting background clutters. More recently, Kuang et al. [33] proposed a deep learning method for single IR image enhancement. A fully convolutional neural network was used to produce images with enhanced contrast and details while a Conditional Generative Adversarial Networks (CGAN) was incorporated into the optimisation framework to enhance the contrast and details meanwhile avoiding amplifying background noise.

Most of the aforementioned deep learning based methods require the associated RGB information to enhance the targeted IR images in the testing stage, which limits their applications on some scenarios where the RGB camera is not available, for example, active thermography in NDT. Inspired by [33,34] for image-to-image translation and cascade networks for single IR image enhancement, we introduce a novel framework that provides a cross-imaging-modality edge learning (CIMEL) capability to achieve IR edge enhancement without amplifying environment noise. Different from other methods, during the testing stage, only IR images are required for the proposed method. The original IR image is utilised to lead the updating of the low-frequency discriminator while the potential edge relationship between the visual and infrared domain is learned through the high-frequency discriminator within the framework.

Compared with the existing IR edge/detail enhancement approaches mentioned above, the proposed framework has the following novelties. Firstly, it investigates a CIMEL strategy which delivers enhanced edges for IR images based on the correspondence information from digital images. The framework directly considers the edge information in the visible spectrum with the correspondence infrared occasion into the downstream training of GAN, which is an essential and critical feature for achieving the desired performance. Secondly, the network is trained by taking into account a pair of carefully designed discriminators and several loss functions. This strategy allows the learning procedure of the visual edge information to be explicitly evaluated within another domain. Thirdly, our proposed framework provides a highly dynamic learning mechanism which adopts visible and infrared images for training while accepting infrared images only for testing. Taking the active thermography, for example, during the model training process, the RGB camera can be used to collect data along with the thermal camera to inspect simulation samples (e.g., flat-bottom holes), where the physical boundary of artificial defect/damage can be measured using the RGB camera. During the model testing or real applications, the RGB camera is not required anymore and the only required input is thermal images. Then the pre-trained model can be used to better estimate the physical boundary of real damage.

## 2. Methodology

**Figure 1:**
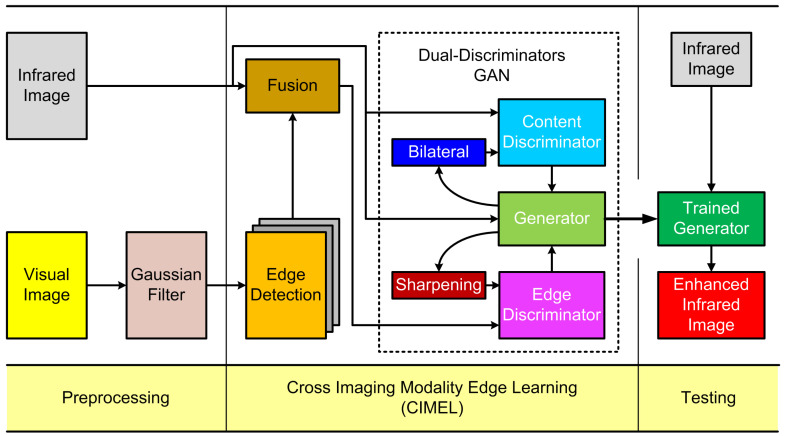
The proposed CIMEL framework to enhance edges in IR images by acquiring knowledge from images in the visible spectrum.

The overall scheme of the proposed framework is illustrated in Figure 1. First of all, a Gaussian Blurring filter [35] is applied on the visual image, which aims to avoid introducing extra noise in the next edge detection step. After edge detection, the edge information from the visual image is fused with the raw IR image. Then, a dual-discriminator (edge and content discriminators) is utilised to guide the CIMEL procedure of the generator in high and low frequencies respectively. During the testing phase, the edge enhanced IR image can be obtained by applying the final model from the generator on the testing IR image only. In the following sections, we first describe the proposed CIMEL idea, then detail the key components within the framework, namely dual-discriminator Conditional Generative Adversarial Networks (CGAN) and loss functions.

### 2.1. Cross Imaging Modality Edge Learning (CIMEL)

Inspired by the image-to-image translation, this study aims to acquire certain information that establishes the specified relationship of attributes (e.g., edge for this study) between different imaging modalities. Through combining this knowledge/information with a learning procedure, translated from visual images and applied on IR images, we introduce a dual-discriminator CGAN based framework that iteratively enhances IR edge by inferring from the visual domain. The main challenges of such a learning approach are presented as follows. Firstly, no extra edge information should be fabricated into the enhanced IR image; Secondly, the content information (low-frequency) of the enhanced IR image should be consistent with the original one while the exclusive information in the visual image should not be transferred; Thirdly, the environmental noise should not be amplified through the learning platform.

In order to overcome the above challenges, we propose a CIMEL framework, which can be expressed as:

(1) $$\begin{array}{c} Out_{IR}\left(x\right)=\overset{\mathrm{Edge}\mathrm{inferring}}{\overset{︷}{F_{e}\left({In}_{IR}\left(x\right),E_{VIS}\left(x\right)\right)}}+\overset{\mathrm{Content}\mathrm{consisting}}{\overset{︷}{F_{c}\left({In}_{IR}\left(x\right),C_{IR}\left(x\right)\right)}}, \end{array}$$

where *x* denotes the pixel coordinate in the IR image, $Out_{IR}\left(x\right)$ is the final enhanced IR image, $In_{IR}\left(x\right)$ presents the input IR image, $E_{VIS}\left(x\right)$ stands for the edge information derived from the visual image, while $C_{IR}\left(x\right)$ contains the non-edge/content information. $F_{e}$ describes the procedure that enhances the IR edge information by considering the detected edges in the visual image, while $F_{c}$ depicts a principle that the content of the edge enhanced IR image should be consistent with the content of the original IR image.

#### 2.1.1. Preprocessing

Denoising and edge enhancement is a pair of contradictions. It is unconscionable to directly enhance IR images with background noise since edge and noise information are both in the high-frequency domain. In order to extract the ideal edge information, Gaussian Blurring (GB) is used for denoising ahead of edge detection to avoid amplifying the environmental noise. It should be noted that, ideally, the denoising method should be chosen appropriately according to the selection of the following edge detection method.

#### 2.1.2. Edge Detection

Edge knowledge is a significantly important factor in this framework, which defines the knowledge to be learned. The higher quality of edge extraction means better learning target, which leads to better enhancement effect. In this study, the edge information from the visual image is detected through a deep learning-based method, Holistically-Nested Edge Detection [36] (HED). HED produces better edges than the classical methods. However, it should be noted that the optimal edge detection is subjective to the purpose of enhancement, the investigation of which is not the scope of this study. The proposed framework can accommodate different types of edge detection methods.

#### 2.1.3. Fusion

The raw IR image and edge information from the visual image are fused and then serve as one of the inputs of the edge/high-frequency discriminator, which guides the CIMEL process. The fusion expression applied is written as:

(2) $$\begin{array}{c} dst=src_{1}\times a+src_{2}\times b+c, \end{array}$$

where dst is the produced fused image, $src_{1}$ and $src_{2}$ are the input images, denoting the raw IR image and the edge image respectively; *a* and *b* are parameters describing the weight of two input images, which were chosen as 0.6 and 0.4 respectively in this study to reach the optimal visualisation; *c* is a constant which was set to 0 in this study. It should be noted that the selection of these parameters only affects the visualisation of enhanced images, but not the quantitative analysis in this paper.

### 2.2. Dual-Discriminator Conditional Generative Adversarial Networks (CGAN)

#### 2.2.1. Structure

To enhance IR edges through the information derived from the visual domain, we propose a dual-discriminator CGAN to achieve the aim of CIMEL. A U-Net [37] “links” style is adopted for the generator to produce enhanced edges and avoid relatively blurry effects. In the meantime, two discriminators based on PatchGAN [34] architecture are employed to supervise the learning procedure of the generator in both low frequency (content) and high frequency (edge) domains. Bilateral Filter [38] (BF) and sharpening operators are deployed as intermediate links between the generator and discriminators to extract and transfer different frequency information.

The basic idea of BF is to consider both spatial and similarity information of the image to be filtered which can be considered as Equation (3):

(3) $$\begin{array}{c} h\left(x\right)=k^{-1}\left(x\right)\;\int \int \;f\left(\epsilon \right)c\left(\epsilon ,x\right)s\left(f\left(\epsilon \right),f\left(x\right)\right)d\epsilon \end{array}$$

where k(x) is the normalization function:

(4) $$\begin{array}{c} k(x)=\;\int \int \;c(\epsilon ,x)s(f(\epsilon ),f(x))d\epsilon . \end{array}$$

h(x) is the filtered result, f(ϵ) is the input image, *x* standards the image coordinate, ϵ describes the neighbour of *x*, *c* represents low-pass filter whilst *s* denotes the range filter.

BF has been proven to have a superior performance to reduce high-frequency noise meanwhile preserving the true edges. Therefore, it is an appropriate filter to ensure the consistency of low-frequency information with the original IR image that usually has relatively less high-frequency noise. A BF is applied within the framework to post-process the image produced by the generator to ensure the consistency of low-frequency information with the original IR image through the guidance of the content/low-frequency discriminator.

A sharpening operation is also applied for the generated image from the generator to assist with emphasizing edge/high-frequency information within the CIMEL procedure. However, it should be noted that the optimal sharpening operation is subjective to the purpose of the enhancement. We applied an arbitrary linear filter to the image and the following sharpening kernel was utilized in this work:

(5) $$\left\{\begin{array}{ccc} 0 & -1 & 0\\ -1 & 5 & -1\\ 0 & -1 & 0 \end{array}\right\}.$$

Intrinsically, we prefer the generator to enhance edge/high-frequency information more than content/low-frequency information without amplifying the noise signals. Therefore, two knowledge extractors (BF and sharing operations) at the downstream of the generator play an essential role in transferring different frequency information from the generator to the discriminators during the learning process. In order to achieve convergence effectiveness, we normalize the intensity value of the input image (the raw IR image) to [0,1], and then feed it into the generator. The intermediate feature maps obtained from each layer spread within the generator until reaching the final layer to produce the output image. Different from the original GANs that only use a random noise as the input, the CGANs-style generator in this study requires the raw IR image as another input for the labelling purpose to ensure enhanced edge/high-frequency information whilst preserving content/low-frequency information.

**Figure 2:**
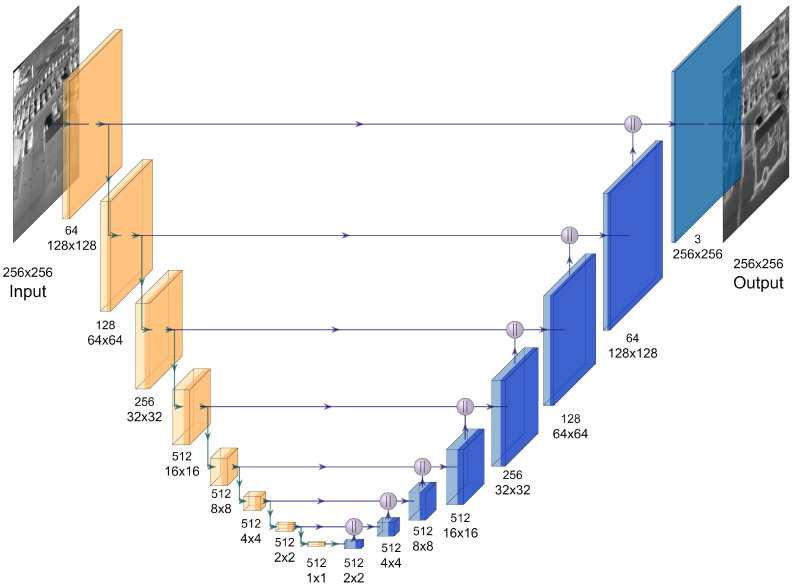
The architecture of the proposed generator. Yellow blocks are downsampling layers and deep blue blocks are upsampling layers.

As shown in Figure 2, the input of the generator is the original IR image and the output is the edge enhanced result. Firstly, the input image is resized to 3×256×256, then 8 convolution layers are deployed in the downsampling stage. The raw images in the selected data have different sizes. To simplify the network design, we assume the input image size is 256×256. In each layer, 4×4 convolution kernels are applied with the stride of two, followed by a batch normalization layer and the LeakyReLU activation function (represented by orange blocks). At the upsampling stage, eight deconvolutional layers are deployed with the stride of two followed by dropout and activation function. The first seven layers (represented by the blue blocks) use the ReLU activation while the last layer (represented by the cyan block) uses the tanh activation function. It should be noted that dropout is applied only in the first three upsampling layers. Concatenate layers are also applied between different layers in the upsampling stage to produce results by directly connecting with layers in the downsampling stage.

#### 2.2.2. Content-Edge Discriminators

**Figure 3:**
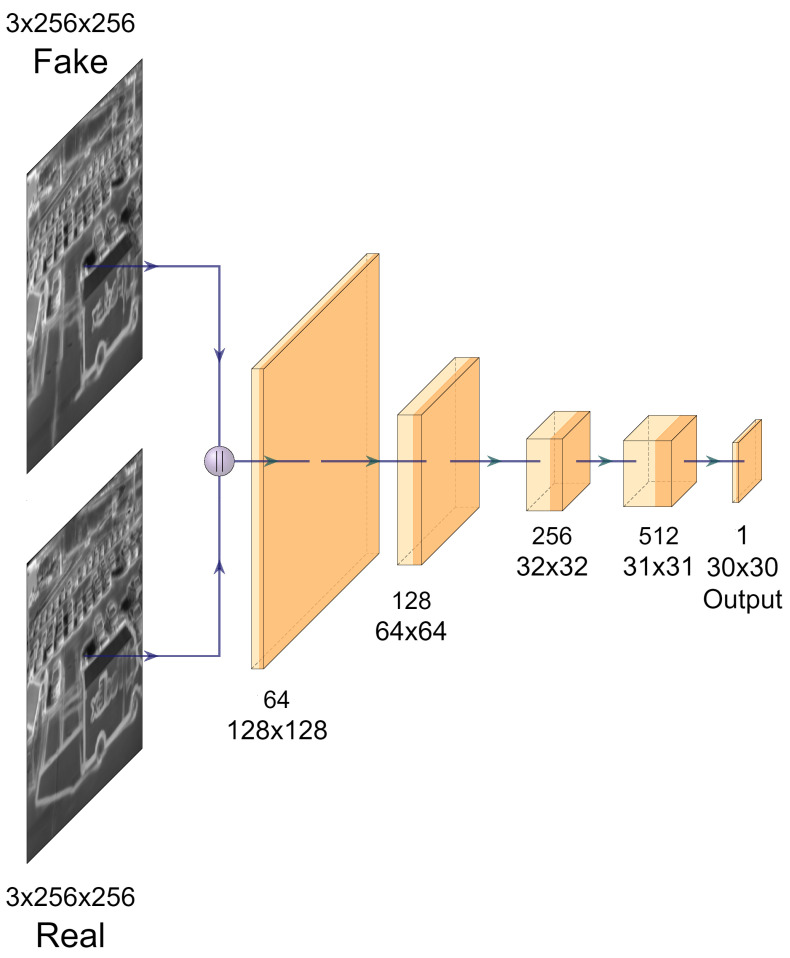
The proposed discriminator structure, which is used for both discriminators.

Due to relatively low contrast and blurred details, as typical characteristics of IR images, one discriminator is difficult to enhance edge regions adaptively while preserving the content texture globally. On the other hand, in addition to a suitable post-denoising operation, edges and content can be conveniently separated from each other within the frequency domain. In CIMEL procedure in two different frequency domains respectively, both adopting PatchGAN [34] for real/fake discrimination. It should be noted that the structure of both discriminators is same and it can be illustrated by Figure 3. In a single discriminator, the input image from the generator is concatenated with the target image and downsampled into 256×32×32 by applying by three layers with a 4×4 kernel size, followed by a Zeropadding layers to increase the size to 256×34×34. After that, a convolution layer with a 4×4 kernel and stride of one is applied, followed by a batch normalization and LeakyReLU activation. Another Zeropadding layer is applied before the last convolution layers (4×4 kernel size and stride of 1). Such a dual-discriminator structure assists the generator to produce an edge enhanced IR image following the low frequency information of the original one, which is critical in avoiding content missing or fake edges.

Intuitively, we utilize the same structure with different loss functions for the two discriminators, which guides the generator to synthesize the edge-enhanced IR image by tackling low and high frequencies information within the same scene in different ways. The Mean Square Error (MSE) loss is used for the low-frequency discriminator while the negative log-likelihood (NLL) loss is used in the high-frequency discriminator. Furthermore, initial thresholds (2.3 for HED and 2.0 for LoG) are selected for both discriminators during each step of the generator training process. Meanwhile, the proposed framework has an iterative serial structure where the generator’s output is judged by the content discriminator $D_{c}$ and edge discriminator $D_{e}$ one by one.

The loss function of the framework can be described as:

(6) $$\begin{array}{cc} G^{*}= & arg\min_{G}\max_{D_{c},D_{e}}L_{CGAN}\left(G,D_{c}\right)+\\ \; & \alpha L_{MSE}\left(G\right)+L_{CGAN}\left(G,D_{e}\right)+\beta L_{L_{1}}\left(G\right), \end{array}$$

where α and β are parameters, $L_{MSE}\left(G\right)$ is MSE loss, $L_{L_{1}}\left(G\right)$ is L1 loss, $L_{CGAN}\left(G,D_{c}\right)$ and $L_{CGAN}\left(G,D_{e}\right)$ are the loss of CGANs for $D_{c}$ and $D_{e}$ respectively, written as:

(7) $$\begin{array}{cc} L_{CGAN}\left(G,D\right)= & E_{x,y}\left[logD\left(x,y\right)\right]+\\ \; & E_{x,z}[log\left(1-D\left(x,G\left(x,z\right)\right)\right]. \end{array}$$

### 2.3. Loss Functions Design

The loss function of $D_{c}$ and $D_{e}$ can be described as:

(8) $$\begin{array}{cc} G^{*}= & arg\min_{G}\max_{D_{c}}\max_{D_{e}}+L_{cGAN}\left(G,D_{c}\right)+\\ \; & \alpha L_{MSE}\left(G\right)+L_{cGAN}\left(G,D_{e}\right)+\beta L_{L_{1}}\left(G\right), \end{array}$$

where α and β are parameters, $L_{MSE}\left(G\right)$ is Mean Squared Error loss, $L_{L_{1}}\left(G\right)$ is L1 loss, $L_{cGAN}\left(G,D_{c}\right)$ and $L_{cGAN}\left(G,D_{e}\right)$ the objective of a conditional GANs, which is:

(9) $$\begin{array}{cc} L_{cGAN}\left(G,D\right)= & E_{x,y}\left[logD\left(x,y\right)\right]+\\ \; & E_{x,z}[log\left(1-D\left(x,G\left(x,z\right)\right)\right]. \end{array}$$

In the early stage of model training, if the error is large, MSE will penalize the large error and the effect will be more significant. However, when the error in the subsequent training phase is small, MSE is not an appropriate choice. Therefore, we use the MSE loss in the low-frequency discriminator initially, and in the next high-frequency discriminator, the NLL loss is used to ensure the final accuracy of the model.

**Figure 4:**
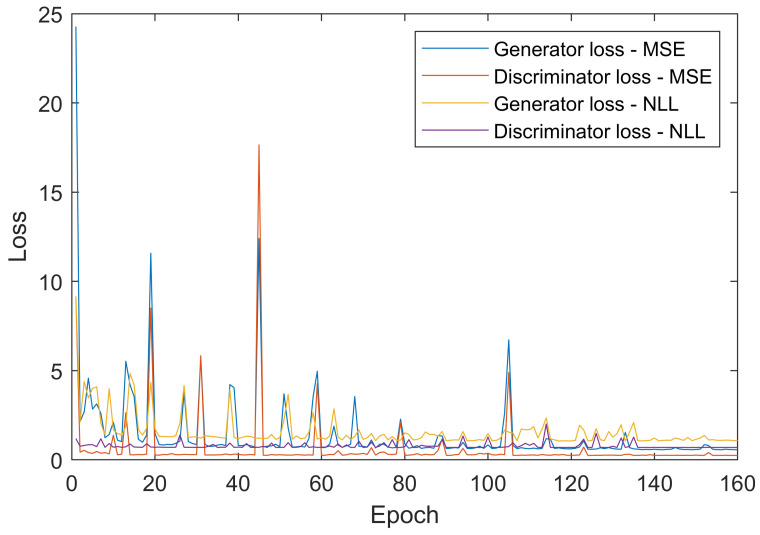
The loss curves of MSE and NLL for both discriminator and generator during training when applying the HED detection method.

The result of comparing the influence of the two loss functions for two different discriminators is shown in Figure 4. This result was produced from two training by applying MSE and NLL losses only for one discriminator. In early training, before epoch 20, when the system shows a remarkable fluctuation, the system reacts more strongly by applying MSE loss. In the middle of the training, around epoch 100, the relative MSE loss was remarkably higher than the NLL loss, which suggests that the power of penalizing MSE loss is larger than that of the NLL loss in early training. While after epoch 120, the response of applying the NLL loss still works better than that of applying the MSE loss.

## 3. Experiments and Results

### 3.1. Dataset and Implementation Details

In order to achieve the novel ability of CIMEL, images paired with the correspondence occasion are essential for the training procedure of the framework. We employed all the video sets within the INO video analytics dataset [39] to demonstrate the performance of the proposed method. An outdoor platform, called VIRxCam, was used to capture the two geometrically aligned streams. Firstly, we sampled the IR and visual video sets with the same frequency respectively which provides 4876 corresponding IR-VIS image pairs. We mixed all nine scenes and randomly selected 3901 pairs (80%) for training while the rest 975 pairs (20%) for testing. All these images were converted to PNG format and resized to 256×256 pixels for convenience.

The framework was implemented in Pytorch using a batch size of 1. The filter weights of each layer within the framework were initialized with a Gaussian initializer with zero mean and standard deviation of 0.02, and disabled the bias. The ADAM optimizer was used with default parameters and a fixed learning rate $2e^{-4}$ for the network optimization. The whole training processing took 10 h on an RTX 2070 Super GPU.

### 3.2. Evaluation

**Figure 5:**
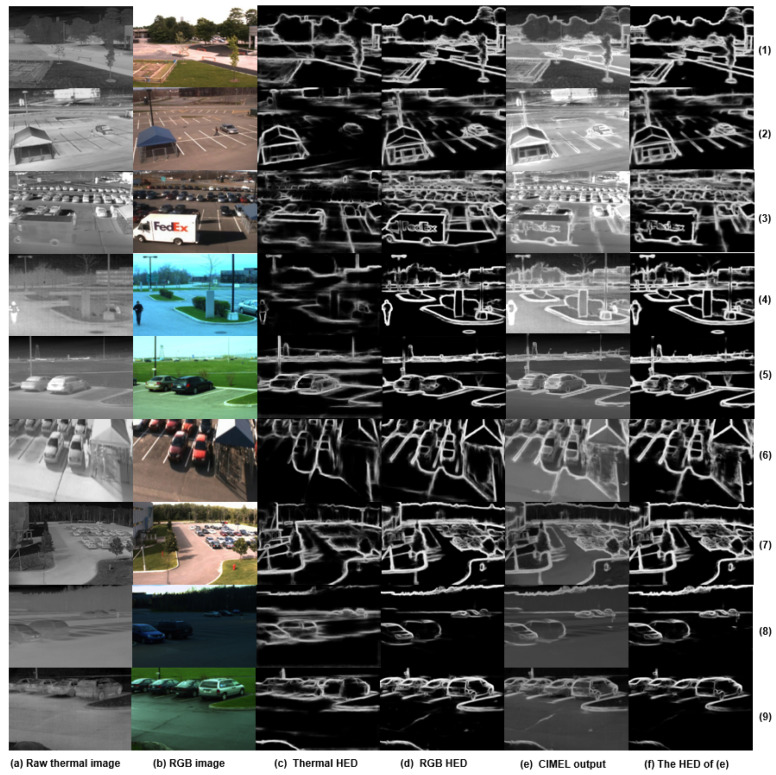
Results of 9 scenes using the proposed CIMEL framework where HED is used for edge detection.

The results of nine videos using the HED edge detection algorithm are demonstrated in Figure 5, where the first column shows the original IR images, the second column is the original visual images, the third to fourth columns are the detected edges using HED for IR and visual images respectively, the fifth is the output results, and the last column highlights the enhanced edge in the outputs.

**Figure 6:**
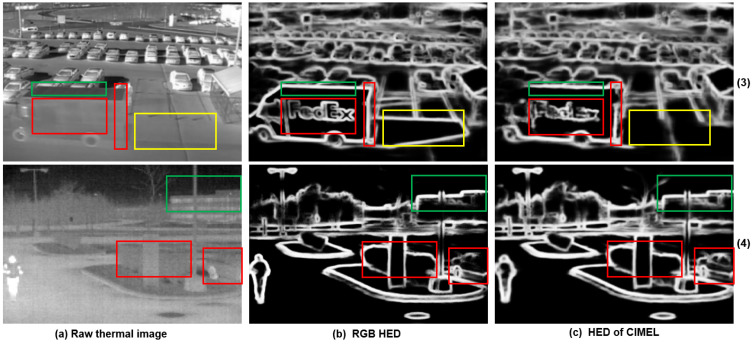
Three types of edges from the HED algorithm: Edges invisible in the IR spectrum but visible in the corresponding visual image (yellow rectangle); Edges weak in the IR spectrum but strong in the corresponding visual image (red rectangle); Edges which are strong in both IR and visual images (green rectangle).

**Figure 7:**
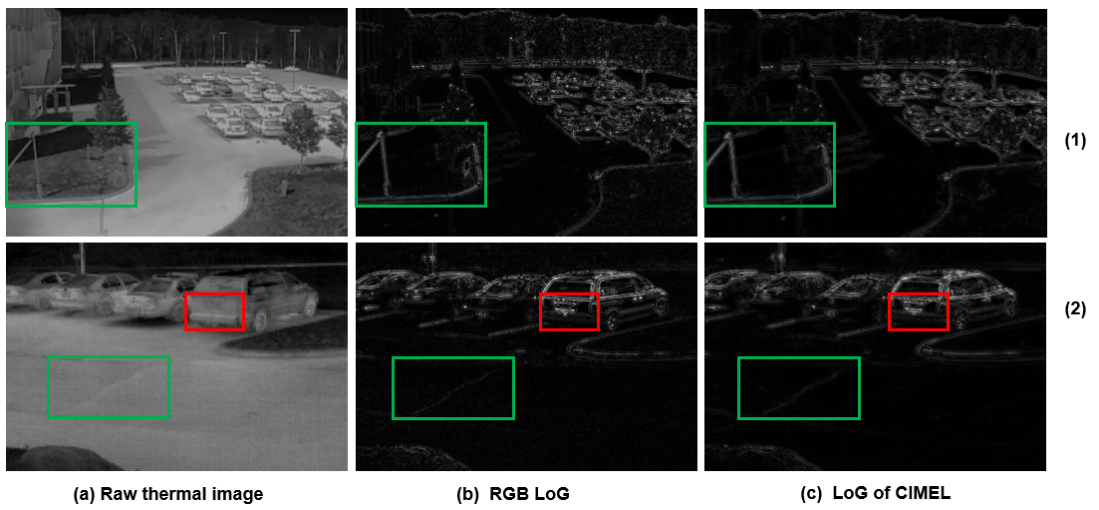
Two types of edges from the LoG algorithm: Edges weak in the IR spectrum but strong in the corresponding visual image (red rectangle); Edges which are strong in both IR and visual images (green rectangle).

In order to specifically depict the edge enhancement effect of the CIMEL framework, edges are divided into four categories for discussion:

(1) Edges which are invisible in the IR spectrum but visible in the corresponding visual image. As demonstrated by the yellow rectangle in the first row of Figure 6, the proposed CIMEL framework does not produce artefact edges of vehicle shadow which appear in the visual image, as shown in the first row of Figure 6b. This kind of edge should not appear in the enhanced IR images as the shadow does not create a difference of temperature;
(2) Edges which are weak in the IR spectrum but strong in the corresponding visual image. For the text on the truck body (‘FedEx’), illustrated by the red rectangle of Figure 6 in scene 3, the edge has been significantly enhanced in the output. Although this information is barely visible in Figure 6a, in theory, different colour coating materials absorb infrared radiation with different degrees and can be recognised by a thermal imaging camera, although the signal is weak. For scene 3, the vertical edge of the van is also enhanced in Figure 6c. For scene 4, the boundary of bush and vehicle is significantly enhanced in the output. This vehicle is barely visible in the original thermal but becomes obvious in the output images, which could have wide application in surveillance. In scene 2 of Figure 7, where the LoG edge detection method is used, the car registration plate can not be identified clearly in the raw thermal image. The enhanced IR image has a much better view of this information contributed by CIMEL. This kind of recovery is unlikely achieved by other edge enhancement methods without the contribution of visual images;
(3) Edges which are strong in both IR and visual images. It can be clearly observed that such edges, indicated by the green rectangles in Figure 6, are well preserved, for example, the top horizontal edge of the vehicle in scene 3, the building in scene 4. Such edges are also demonstrated in Figure 7 in the area of the flowerbed in scene 1 and the road in scene 2.

To quantitatively evaluate the performance, Structural Similarity Index Measure (*SSIM*), Peak signal-to-noise ratio (*PSNR*) and Recall were employed to measure the similarity between the edges in the original IR image and visual image, and the similarity between the edges in the enhanced IR image and visual image. *PSNR* can be calculated by:

(10) $$\begin{array}{c} PSNR=20\times log_{10}\frac{MAX_{I}}{\sqrt{MSE}}, \end{array}$$

where $MAX_{I}$ is the max possible value of images (256 in this study), MSE is Mean-Square Error calculated by:

(11) $$\begin{array}{c} MSE=\frac{1}{m\times n}\sum_{i=1}^{m}\sum_{j=1}^{n}{\left[I\left(i,j\right)-K\left(i,j\right)\right]}^{2}, \end{array}$$

where m×n is the size of the image, *I* is the edge of the original or enhanced IR image and *K* is the edge of the visual image. SSIM can be calculated by:

(12) $$\begin{array}{c} SSIM=\frac{\left(2\mu_{I}\mu_{K}+c_{1}\right)\left(2\sigma_{I,K}+c_{2}\right)}{\left({\mu_{I}}^{2}+{\mu_{K}}^{2}+c_{1}\right)\left({\sigma_{I}}^{2}+{\sigma_{K}}^{2}+c_{2}\right)}, \end{array}$$

where μ and σ describe the average and variance respectively, $c_{1}$ and $c_{2}$ are constants for stabilising. SSIM, focusing on the similarity of structure, is more appropriate to evaluate the edge enhancement, while PSNR can be evaluated the general quality of the generated images. After the edge image is computed, the Otsu’ method is used to binarise the image automatically. The True Positive (TP) value and False Negative (FN) value are calculated by comparing the binarised RGB edge image and the corresponding binarised raw thermal edge image or the enhanced thermal edge image. Recall is then calculated by TP/(TP + FN) for the cases before and after CIMEL, respectively.

The results before and after CIMEL using HED and other two classic edge detection methods, Laplacian of Gaussian (LoG) and Canny, are shown in Table 1. The results were calculated by averaging the outputs of the 975 testing images. It can be observed that SSIM has been improved significantly for both HED and LoG (from 0.493 to 0.854 and 0.390 to 0.785, respectively) while Canny has a relatively low improvement (from 0.406 to 0.466). This is due to edges detected by Canny being binary-based and have artefacts to produce close contours. For PSNR, the proposed CIMEL enhanced the edges from LoG and HED significantly, but not for the edges from Canny for a similar reason to that mentioned above. For Recall, the proposed CIMEL enhanced the edges for all three edge detection methods significantly.

**Figure 8:**
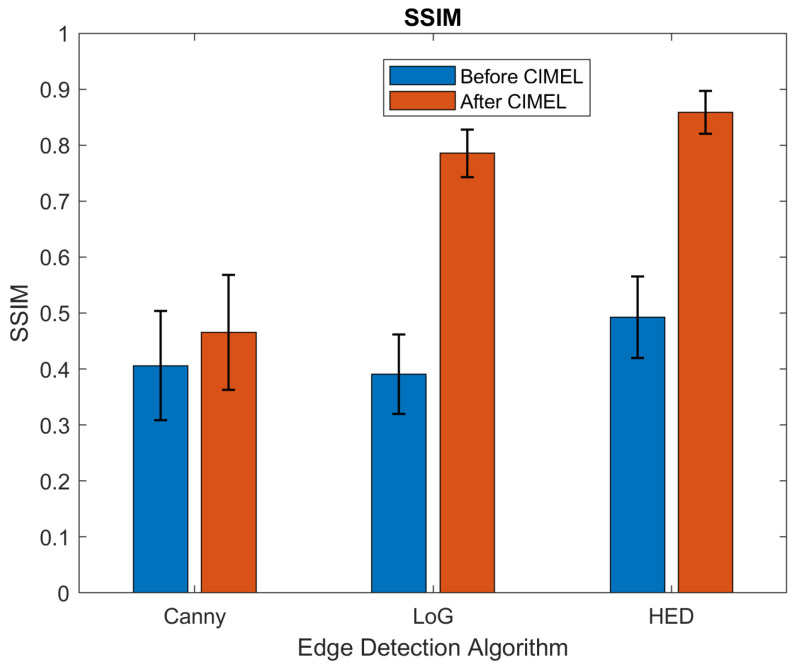
Results of SSIM before and after CIMEL for three edge detection methods.

**Figure 9:**
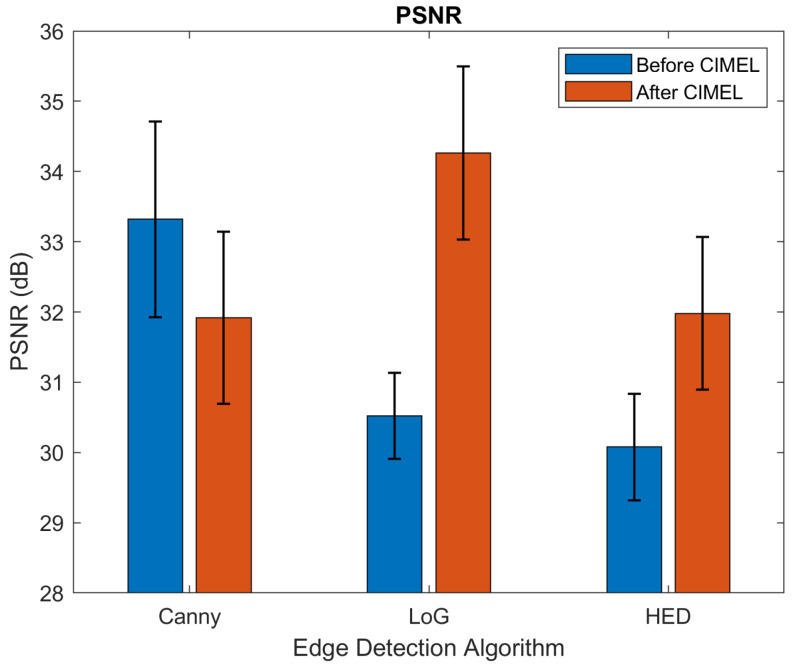
Results of PSNR before and after CIMEL for three edge detection methods.

**Figure 10:**
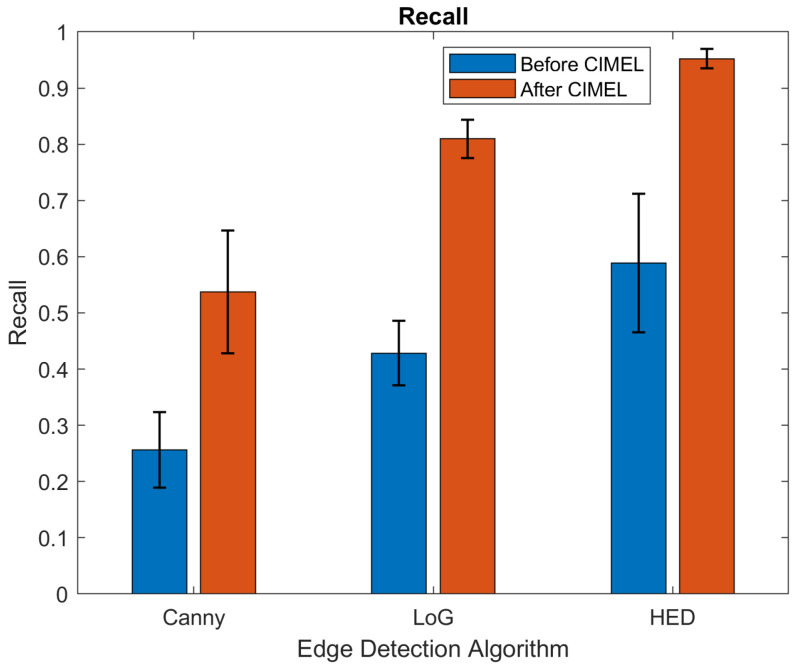
Results of Recall before and after CIMEL for three edge detection methods.

The detailed statistical results can be found in Figure 8, Figure 9 and Figure 10. It can be observed that the results after CIMEL in SSIM have smaller variations than those before CIMEL, particularly for LoG and HED. This observation suggests that CIMEL has a consistent good performance across different scenes in terms of structural similarity. For PSNR, it can be observed that the results after CIMEL have larger variations than those before CIMEL, particularly for LoG and HED. This suggests that, although CIMEL enhances the visual edges in the thermal image, the improvement in the pixel level has a large variation across different scenes. It should be noted that SSIM is more closely related to the human visual system as it extracts useful information as luminance, contrast and structure. For Recall, the improvement of the average value is much more obvious with an almost 100% improvement for all three edge detection methods. Similar to SSIM, the results after CIMEL in Recall have smaller variations than those before CIMEL for LoG and HED.

## 4. Conclusions

Addressing the strong demand of thermal image enhancement, this paper reports a novel Conditional Generative Adversarial Networks (CGAN) based approach to achieve this aim by inferring edges in thermal images from edges in visual images. To treat the high and low frequencies information separately, we introduced a dual-discriminator focusing on edges and content/background respectively. With such a model, edges of thermal images can be enhanced without the inputs of visual images in the testing stage. The qualitative results demonstrated that the proposed method can effectively enhance weak edges in IR images that are strong in visual images. Meanwhile, edges that are strong in both IR and visual images are preserved. The artefact edges, which should not appear in IR images, are compressed.

It should be noted that CIMEL is not a fusion method as only one imaging modality is required during the testing stage. It is an attempt to enhance features, which are weak in one imaging modality due to the physical limitation through learning from these features, which are strong in another imaging modality. This is the main novelty of this research. The direct application of the proposed method is active thermography in NDT. Active thermography detects the surface and subsurface defects based on the temperature decay profile, where the accuracy of defect measurement is affected by the low spatial resolution of IR cameras and blurred boundaries caused by heat conduction. The proposed CIMEL technique can learn the surface defect measurement using digital cameras which usually have a much higher spatial resolution and produce sharper edges, to enhance the defect measurement accuracy of thermography. Additionally, the proposed solution can be extended to other applications, such as edge enhancement for digital images benefiting from the corresponding thermal images. This will be particularly useful for human detection and tracking under insufficient illumination in the surveillance application. The methodology for such an application is almost the same and the only difference is that the feeds of digital images and thermal images should be swapped. This paper is a proof-of-concept and the full exploration of this technique requires further study.

This produced model is limited by the limited number of videos in the dataset. Although there is no overlap of the training images and testing images, all nine videos are used for training. The trained model in this paper will likely have relatively poor performance on data for other scenes. In future work, we will extend the database and test the model’s performance on different scenes.

## Figures and Tables

**Table 1:** Average SSIM, PSNR and Recall between edges or IR images and visual images before/after enhancement.

Edge	SSIM	PSNR	Recall
LoG	0.39/0.79	30.52 dB/34.27 dB	0.43/0.81
Canny	0.41/0.47	33.32 dB/31.43 dB	0.26/0.54
HED	0.49/0.85	30.08 dB/31.98 dB	0.59/0.95

## Data Availability

We use the public dataset, the link of which has been provided in the reference list.
